# CTRP9 Mitigates the Progression of Arteriovenous Shunt-Induced Pulmonary Artery Hypertension in Rats

**DOI:** 10.1155/2021/4971300

**Published:** 2021-11-10

**Authors:** Hua Guan, Xiaofeng Yang, Tao Shi, Yongjian Zhang, Aoqi Xiang, Yongxin Li

**Affiliations:** ^1^Shaanxi Key Laboratory of Ischemic Cardiovascular Diseases & Institute of Basic and Translational Medicine, Xi'an Medical University, Xi'an, 710021 Shaanxi, China; ^2^Department of Pathogenic Microbiology and Immunology, School of Basic Medical Sciences, Xi'an Jiaotong University, Xi'an 710061, Shaanxi, China; ^3^Department of Cardiovascular Surgery, The First Affiliated Hospital of Xi'an Jiaotong University, Xi'an, 710061 Shaanxi, China

## Abstract

The present study is aimed at investigating the molecular mechanism of C1q/TNF-related protein 9 (CTRP9) and providing a new perspective in arteriovenous shunt-induced pulmonary arterial hypertension (PAH). PAH was established by an arteriovenous shunt placement performed in rats. Adenovirus(Ad)-CTRP9 and Ad-green fluorescent protein viral particles were injected into the rats through the tail vein. Following 12 weeks, the mean pulmonary arterial pressure (mPAP) and right ventricular systolic pressure (RVSP) were measured and morphological analysis was conducted to confirm the establishment of the PAH model. The systemic elevation of CTRP9 maintained pulmonary vascular homeostasis and protected the rats from dysfunctional and abnormal remodeling. CTRP9 attenuated the pulmonary vascular remodeling in the shunt group by decreasing the mPAP and RVSP, which was associated with suppressed inflammation, apoptosis, and extracellular matrix injury. In addition, CTRP9 dramatically increased the phosphorylation of AKT and p38-MAPK in the lung tissues of shunt-operated animals. These findings suggest a previously unrecognized effect of CTRP9 in pulmonary vascular homeostasis during PAH pathogenesis.

## 1. Introduction

Pulmonary arteriole remodeling occurs in patients with pulmonary arterial hypertension (PAH), and as a result, pulmonary vascular resistance gradually increases, leading to right heart failure and premature death [1]. The pathological characteristics of PAH, namely, pulmonary artery endothelial cell dysfunction, smooth muscle hyperplasia and hypertrophy, and vascular stenosis, result in pulmonary arterial blood flow reduction and pathological changes [2–4]. To date, the pathological mechanism of PAH has not been fully elucidated. The injury caused to pulmonary artery endothelial cells by continuous high blood flow shock is one of the possible mechanisms responsible for the formation of PAH [5, 6].

Adiponectin is synthesized and secreted into the extracellular matrix by adipocytes and exerts its physiological effects by activating cell receptors. A previous study indicated that adiponectin exhibits anti-inflammatory, antiatherosclerotic, and metabolic effects [7]. C1q/TNF-related protein (CTRP) family members contain collagen-like and globular C1q-like domains, which are highly similar to those of adiponectin [8]. Among the CTRPs, the amino acid sequence of CTRP9 shares the highest homology with adiponectin and it is also abundantly expressed in adipose tissues [9]. Animal experiments have shown that CTRP9 exerts a protective effect on diseases of the cardiovascular system. By inhibiting the apoptosis and inflammation of cardiomyocytes, CTRP9 reduces myocardial damage via the regulation of the function of smooth muscle cells (SMCs) and the alleviation of the pathological progression of vascular remodeling [10]. In addition, it has been shown that overexpression of CTRP9 can significantly reduce the high-fat diet-induced formation of atherosclerotic plaques [11]. It has also been shown that CTRP9 plays an important role in the phagocytosis of lipids by macrophages during the formation of foam cells [12]. By inhibiting this process, CTRP9 can prevent endothelial cell injury [12].

Our research team demonstrated previously that CTRP9 ameliorated the pathological progression of PAH by improving the inflammatory response and attenuating endothelial cell survival, as well as by regulating hypoxia-mediated human pulmonary artery SMC proliferation, apoptosis, and migration *in vitro* [13, 14]. However, the mechanism by which CTRP9 protects against PAH *in vivo* is still unclear. In the present study, the data demonstrated that arteriovenous shunt-induced PAH was associated with exacerbation of inflammation, apoptosis, and extracellular matrix accumulation in lung tissues, whereas overexpression of CTRP9 mitigated the pathological progression of PAH by activating the AKT and MAPK pathways. The data provide novel insight into the molecular mechanism of CTRP9 based on PAH animal models.

## 2. Materials and Methods

### 2.1. Animals and Study Protocol

Male, 6-week-old Sprague-Dawley rats, weighing 200-220 g, were purchased from the Laboratory Animal Center of Xi'an Jiaotong University. The animals were housed under clean and specific pathogen-free conditions, and the experimental animal licenses used were the following: SCXK (Shaanxi Huaxi Pharmaceutical Co., Ltd.; 2007-005) and SYXK (Shaanxi Huaxi Pharmaceutical Co., Ltd.; 2003-0026). A maximum of three animals was housed per cage. Food and water were freely available at all times, and the animals were housed at a temperature of 25°C and relative humidity of 50-80% under a normal light cycle.

The animals were randomly divided into four groups: two sham groups treated with adenovirus-green fluorescent protein (Ad-GFP) or Ad-CTRP9 and two shunt groups treated with Ad-GFP or Ad-CTRP9 (*n* = 10 of each group) (note: there were 15 rats in each group for modeling operation. Some rats died during the operation, and the number of rats that survived was more than 10 in each group. When we analyzed the data, 10 rats in each group were randomly selected for analysis. Therefore, the success rate of surgical modeling was 100%, which could ensure that all rats in the experimental group were successfully modeled and survived). In previous experiments, 1 × 10^11^ viral particles of Ad-CTRP9 or Ad-GFP were used as a control. The particles were injected into the tail vein of the sham or shunt rats. Ad-GFP or Ad-CTRP9 were administered every three weeks and injected four times in total. The rats were euthanized by intraperitoneal injection of pentobarbital sodium (150 mg/kg body weight). The research methods and ethical reviews met the requirements of the Institutional Animal Care and Use Committee of Xi'an Jiaotong University. The present study followed the regulations of the Guidelines for the Care and Use of Laboratory Animals published by the US NIH (NIH Publication, 8^th^ Edition, 2011).

### 2.2. Construction of the Adenoviral CTRP9 Vector and Infection of the Cells

A recombinant adenoviral vector encoding CTRP9 (Ad-CTRP9) was constructed according to a previously published method [15]. CTRP9 cDNA was subcloned into the adenoviral shuttle plasmid pAdTrack-CMV. Following sequence confirmation, the recombinant shuttle plasmid was transformed into the BJ5183 competent cell. The recombinant adenovirus was packaged and amplified in HEK293A cells. Following purification, the viral titer was detected by TCID50. An empty adenoviral vector (Ad-GFP) was constructed as a control.

### 2.3. Preparation of the PAH Animal Model

The PAH animal model was established as previously described [16]. Briefly, the rats were weighed and anesthetized by intraperitoneal injection of sodium pentobarbital (60 mg/kg). The abdominal aorta and inferior vena cava were exposed by laparotomy. The free branches of both sides were ligated to block the abdominal aorta and inferior vena cava between the left renal artery and the iliac artery by microscopy. A transverse incision was made on the anterior wall of the abdominal aorta with mini scissors to expose the opposite wall adjacent to the wall of the inferior vena cava. Following placement of the shunt, an 11-0 polypropylene monofilament was used to suture the abdominal aortic wall incision and the terylene thread was loosened and blocked. The pulsation of the inferior vena cava and the swelling of the arterial blood were observed. In the sham group, the anterior wall of the abdominal aorta was sutured immediately following incision. Prior to the formal experiment, the operation of the rats was modeled and the success rate was estimated to 100%. There was no death during the PAH. Therefore, no animal death occurred during the experimental conduct.

### 2.4. Measurement of Mean Pulmonary Arterial Pressure (mPAP)

Left-to-right shunting congenital heart diseases are the common cause of secondary PAH, because increased pulmonary blood flow appears to be a crucial factor in pathogenesis; particular emphasis is given in models of increased pulmonary blood flow that lead to development of pulmonary vascular remodeling. Typically, the PAH model will be formed 12 weeks after the surgery of the aortocaval shunt. In the initial of the surgery operation, there is no difference between the sham and shunt groups. Hence, we measured the mPAP and RVSP at the end of 12 weeks. mPAP was measured in the anesthetized rats as previously described [16]. Sodium pentobarbital (60 mg/kg) was used to anesthetize the rats, which were intubated by directly cutting the trachea. PAP was detected using a 25-gauge needle (heparin infusion) connected to a pressure transducer (model P10EZ; Spectramed). Postmortem examination confirmed that the puncture location was correct. The PowerLab software was used to record the pressure (AD Instruments).

### 2.5. Evaluation of the Right Ventricular Systolic Pressure (RVSP)

Following placement of the arteriovenous shunt, the rats were anesthetized at week 12 with sodium pentobarbital (60 mg/kg). A sternotomy was performed and a Millar catheter (Millar Instruments, KS) was inserted into the right ventricle to measure RVSP, as described in a previous study [17].

### 2.6. Evaluation of Pulmonary Arterial Remodeling

At least five small muscular arteries with complete internal and external elastic lamina were randomly selected from the lung tissue sections of the surviving rats in each group. The sections were embedded in paraffin and stained with hematoxylin and eosin (H&E). The medial thickness (MT), vascular external diameter (ED), and vascular internal diameter of the internal and external elastic lamina were measured for histological analysis. The average medial thickness percentage (MT%) of the pulmonary arterioles was further calculated as follows: MT% = (2 × MT ÷ ED) × 100%.

### 2.7. Assay of Wall Thickness of Pulmonary Arteries

The lungs were prepared as previously described. Sodium pentobarbital (150 mg/kg) was used for rat euthanasia, and the chest and abdominal cavity of the rats were opened. The lung sections were stained with H&E. The morphological changes in the peripheral pulmonary arteries were detected with a digital microscope camera, and the microscopic images were analyzed using the ImageJ software to determine the percentage wall thickness (WT%) and lumen area (LA%). Subsequently, the following formulas were used to calculate the wall thickness ratio of each pulmonary artery: WT% = vessel wall thickness/vessel external diameter × 100% and LA% = vessel lumen area/vessel total area (including external wall of the vessel) × 100%. Six vessels of comparable size per rat were measured (8 rats per group).

### 2.8. Western Blotting

The protein from the lung tissue was extracted, and the concentration was determined by the BCA method. A total of 6 samples were randomly selected from each group to perform western blotting. The proteins were separated and subsequently transferred onto a polyvinylidene fluoride membrane (Thermo Fisher Scientific, Inc.). The membranes were incubated with each primary antibody (Ab) as follows: antimonocyte chemotactic protein 1 (MCP-1), anti-interleukin 6 (IL-6), anti-tumor necrosis factor-*α* (TNF-*α*), anti-IL-18, anti-caspase-8, anti-b-cell lymphoma 2 (Bcl-2), anti-matrix metallopeptidase 2 (MMP-2), anti-MMP-9, anti-AKT serine/threonine kinase 1, anti-p-AKT, anti-p38 mitogen-activated protein kinase (p38-MAPK), anti-p-p38-MAPK, anti-extracellular-regulated protein kinase (ERK), anti-p-ERK, and anti-*β*-actin. The incubations were performed at 4°C overnight, as recommended in the manufacturer's instructions. Following three times washing, the membranes were incubated with horseradish peroxidase-conjugated secondary Ab (1 : 5,000) for 2 h. The blots were developed using a chemiluminescent detection system (EMD Millipore; Merck KGaA).

### 2.9. Reverse Transcription-Quantitative PCR

Total RNA was isolated from the lung tissue with the use of the RNeasy Mini Kit (Qiagen, Inc.) according to the manufacturer's instructions. In the present experiment, we extracted total RNA from lung tissue of 10 samples in each group. Secondly, we evaluated the RNA quality and selected six qualified samples of each group for the follow-up qPCR experiment, and the sample capacity met the statistical requirements. cDNA was reverse-transcribed and synthesized from 1 *μ*g of total RNA and amplified by the Bio-Rad real-time PCR system (Bio-Rad Laboratories, Inc.). The amount of transcripts was quantified, and each sample was normalized according to its *β*-actin content. The real-time PCR primer sequences are shown in Table SI.

### 2.10. Statistical Analysis

All data are expressed as the mean ± SEM. The significance of the differences was assessed by either Student's *t*-test (single comparisons) or one-way ANOVA followed by Tukey's multiple-comparison procedure (multiple comparisons). For unequal variances, the data were evaluated by the Kruskal-Wallis test. *P* < 0.05 was considered to indicate a statistically significant difference.

## 3. Results

### 3.1. Effects of CTRP9 Overexpression on the Physical Characteristics of the Rats

**Figure 1:**
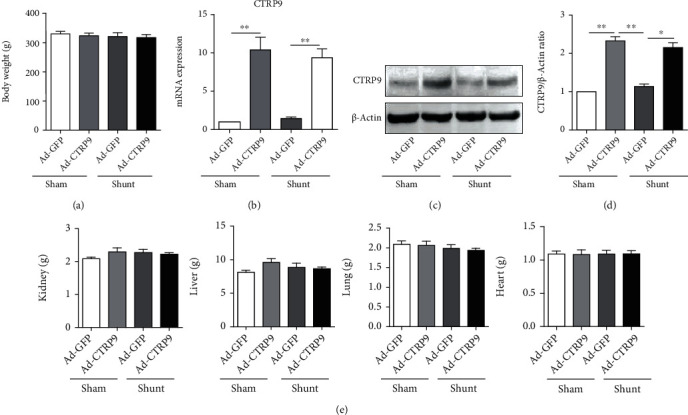
Effects on physical parameters following CTRP9 overexpression in sham and shunt rats. (a) The body weight and the weights of the lung, heart, liver, and kidney of rats. (b) mRNA expression of CTRP9 was measured by RT-qPCR in the shunt and sham groups. (c) Protein expression of CTRP9 was determined by western blotting (*β*-actin was loaded as the control). *n* = 10 of each group. Mean ± SEM. ^∗^*P* < 0.05 and ^∗∗^*P* < 0.01 vs. the Ad-GFP-sham group. RT-qPCR: reverse transcription-quantitative PCR; CTRP9: C1q/TNF-related protein 9; Ad-GFP: adenovirus-green fluorescent protein.

The results indicated no significant differences in the body weight of the animals following shunt-induced PAH (Figure 1(a)). In addition, the weights of the kidney, liver, lung, and heart (Figures 1(a) and 1(e)) were not affected by PAH. Moreover, the mRNA and protein expression levels of CTRP9 were assessed in the lung tissues and the results indicated that following injection of Ad-CTRP9 in the tail veins of the rats, CTRP9 expression was significantly increased in the sham and shunt groups compared with that noted in the Ad-GFP group (Figures 1(b)–1(d)).

### 3.2. Generation of the PAH Model

**Figure 2:**
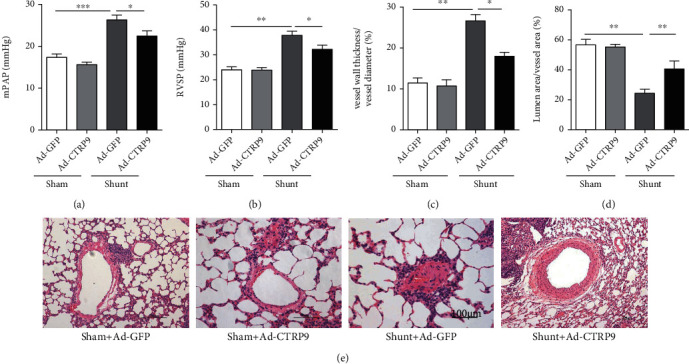
PAH is prevented and attenuated by the administration of CTRP9. (a) The rats in the shunt group exhibited a significantly higher mPAP compared with that of the rats in the sham group at the end of the 12^th^ week. (b) The rats in the shunt group exhibited a significantly higher RVSP compared with that of the rats in the sham group at the end of the 12^th^ week. (c) Quantification of the percentage of wall thickness to vessel diameter. (d) Quantification of the lumen area to vessel total area. (e) Representative images of H&E-stained resistant pulmonary arteries from the different experimental groups. *n* = 10 of each group. Mean ± SEM. ^∗^*P* < 0.05 and ^∗∗^*P* < 0.01 vs. the Ad-GFP-sham group. PAH: pulmonary arterial hypertension; CTRP9: C1q/TNF-related protein 9; mPAP: mean pulmonary artery pressure; RVSP: right ventricular systolic pressure; H&E: hematoxylin and eosin; Ad-GFP: adenovirus-green fluorescent protein.

RVSP and mPAP were elevated in the rats of the shunt group compared with those noted in the rats of the sham group at the 12^th^ week after the tail vein injection of Ad-GFP or Ad-CTRP9. As the duration of the shunt was prolonged, the values of the mPAP and RVSP of the rats in the shunt group were gradually increased. Since increased mPAP and RVSP are observed in the shunt group which could indicate the PAH, the injection of CTRP9 promoted a slight decrease of these parameters. The rats in the shunt group exhibited a significantly higher mPAP than the rats in the sham group (26.3 ± 1.1 mmHg vs. 22.5 ± 1.2 mmHg, *P* < 0.01) at the end of the 12^th^ week following administration of CTRP9 (Figure 2(a)). The rats in the shunt group exhibited a significantly higher RVSP than that of the rats in the sham group (37.9 ± 5.6 mmHg vs. 32.3 ± 4.7 mmHg, *P* < 0.001) at the end of the 12^th^ week following administration of CTRP9 (Figure 2(b)).

H&E staining demonstrated significant vascular SMC proliferation and pulmonary vascular remodeling in the PAH model group compared to those noted in the sham group. In the sham group, the walls of the pulmonary vessels were thin and the intima and media were not readily distinguished (Figure 2(e)). The surgery of the aortocaval shunt caused a significant increase of the tunica media thickness percentage compared to the sham group at the end of the 12^th^ week (Figure 2(c)). The lumen area to vessel area ratio was significantly higher in the shunt group than that noted in the sham group (*P* < 0.05) (Figure 2(d)). The proliferation of the vascular SMCs in the shunt group was more apparent when Ad-GFP was administered compared with the mild proliferation of the vascular SMCs noted in the sham group. Endothelial cell proliferation appeared in the shunt group at the 12^th^ week following the injection of the rats with Ad-GFP (Figure 2(e)).

### 3.3. CTRP9 Mitigates Inflammation, Apoptosis, and Matrix Injury in Lung Tissues

**Figure 3:**
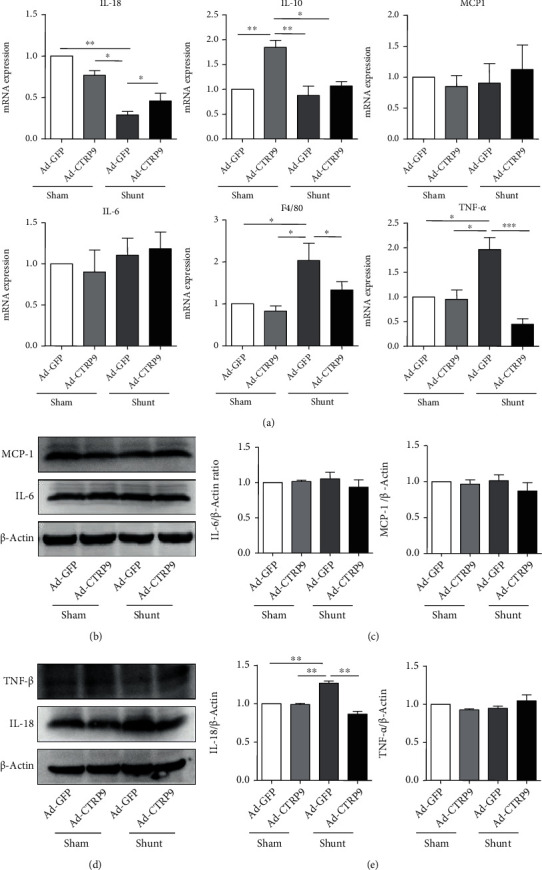
CTRP9 suppresses inflammation *in vivo.* (a) The interleukin 18 (IL-18), IL-10, monocyte chemoattractant protein-1 (MCP-1), IL-6, F4/80, and tumor necrosis factor alpha mRNA expression levels were analyzed by RT-qPCR. (b, d) Injection of the rats with CTRP9 in the sham and shunt groups reduced the protein expression of MCP-1, IL-6, TNF-*α*, and IL-18, as assessed by immunoblot analysis of the total lung tissue. (c, e) Representative western blots of the total lung tissues indicating the induction of the inflammatory markers MCP-1, IL-6, TNF-*α*, and IL-18 following treatment of the rats with CTRP9 in the shunt group (*β*-actin was used as a loading control) *n* = 6 for each group. In (b) and (d), the protein molecular weight of the MCP-1 (18 kDa), IL-6 (21 kDa), TNF-*α* (26 kDa), and IL-18 (22 kDa) was very similar, so they were all originating from the same gel. After exposure of the MCP-1 or TNF-*α*, we striped the primary antibody by the striping solution from the membrane and incubated the TNF-*α* antibody or IL-18 antibody. So, we use the same *β*-actin blot banding as the corresponding lading control from the same membrane. A total of 6 samples were selected from each group to perform RT-qPCR. The data are expressed as the mean ± SEM. ^∗^*P* < 0.05, ^∗∗^*P* < 0.01, and ^∗∗∗^*P* < 0.001 vs. the Ad-GFP-sham group. IL: interleukin; MCP-1: monocyte chemoattractant protein-1; TNF-*α*: tumor necrosis factor alpha; RT-qPCR: reverse transcription-quantitative PCR; CTRP9: C1q/TNF-related protein 9; Ad-GFP: adenovirus-GFP.

To explore the role of CTRP9 in PAH, the lung tissues were isolated and the inflammation-associated gene expression was determined. Following shunt placement, the mRNA expression of the proinflammatory factors TNF-*α* and F4/80 was increased significantly after the administration of Ad-GFP. In contrast to these findings, overexpression of CTRP9 inhibited the mRNA expression of TNF-*α* and F4/80 following shunt operation. A significant decrease in the mRNA expression levels of specific anti-inflammatory factors was noted, including IL-10 and IL-18. However, no significant difference was noted in the mRNA levels of MCP-1 and IL-6 between the shunt and sham groups (Figure 3(a)). IL-18 protein expression was significantly decreased in the shunt group after the injection of CTRP9 (Figures 3(d) and 3(e)). However, MCP-1, IL-6, and TNF-*α* protein expression levels were assessed and the results indicated no significant differences between the sham and shunt groups (Figures 3(b)–3(e)).

**Figure 4:**
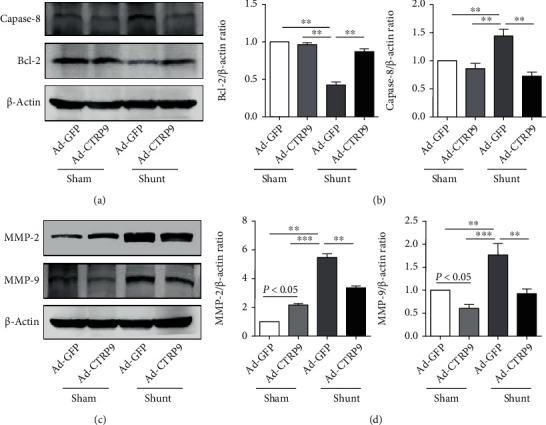
CTRP9 inhibits lung apoptosis and matrix injury. Wild-type rats were injected with Ad-CTRP9 or Ad-GFP, subjected to shunt or sham operation, and samples were obtained at week 12. (a, c) Caspase-8, Bcl-2, MMP-2, and MMP-9 protein expressions were analyzed by western blotting. (b, d) Representative western blots and quantification of the changes noted in caspase-8, Bcl-2, MMP-2, and MMP-9 in the lung tissues of the sham or shunt rats treated with or without CTRP9. *β*-Actin was used as a loading control. The data are expressed as the mean ± SEM. *n* = 6 for each group. A total of 6 samples were randomly selected from each group to perform western blotting. ^∗^*P* < 0.05, ^∗∗^*P* < 0.01, and ^∗∗∗^*P* < 0.001 vs. the Ad-GFP-sham group. CTRP9: C1q/TNF-related protein 9; Ad-CTRP9: adenovirus-CTRP9; Ad-GFP: adenovirus GFP; Bcl-2: b-cell lymphoma; MMP: matrix metallopeptidase.

In addition, the levels of the apoptosis-associated proteins Bcl-2 and caspase-8 were detected by western blotting. Caspase-8 protein expression was significantly increased in the shunt group compared with that noted in the sham group (Figures 4(a) and 4(b)). In addition, CTRP9 inhibited caspase-8 protein expression following shunt placement (Figures 4(a) and 4(b)). Furthermore, Bcl-2 protein expression was significantly increased in the Ad-CTRP9 group compared with that noted in the Ad-GFP group following shunt operation (Figures 4(a) and 4(b)). Following surgery, MMP-2 and MMP-9 protein expression levels were significantly increased (Figures 4(c) and 4(d)). Following administration of Ad-CTRP9, MMP-2 and MMP-9 protein expression levels were significantly decreased (Figures 4(c) and 4(d)).

### 3.4. CTRP9 Promotes AKT Signaling in Lung Tissues

**Figure 5:**
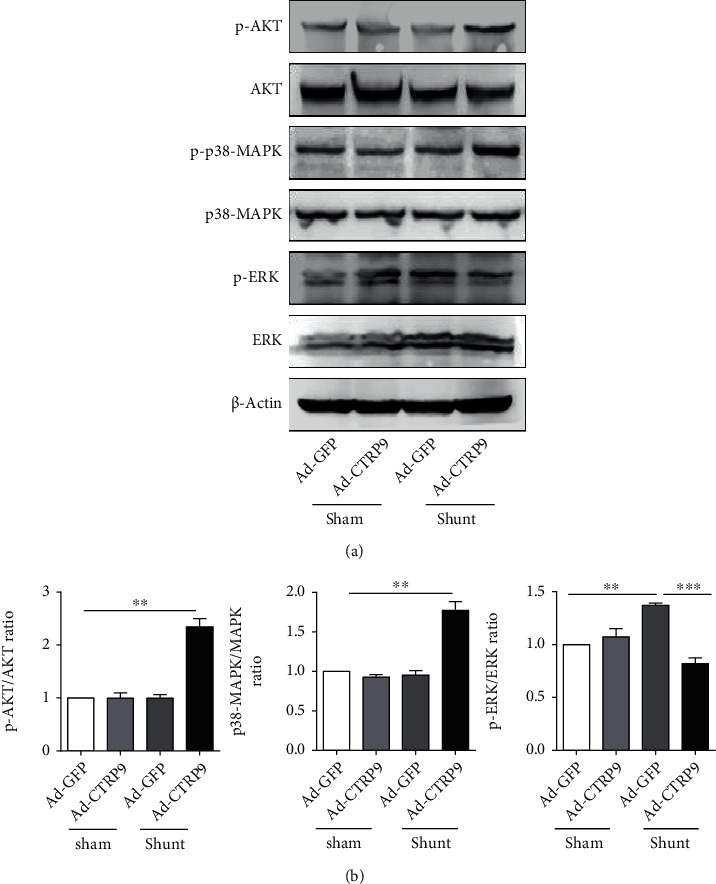
CTRP9 activates AKT signaling to relieve PAH *in vivo*. (a) Following the systemic delivery of Ad-CTRP9 or Ad-GFP, the wild-type rats were subjected to shunt or sham operation. CTRP9 promoted AKT, MAPK, and ERK phosphorylation during PAH. (b) The relative levels of phosphorylated AKT, p38-MAPK, and ERK were quantified using ImageJ and are expressed relative to the *β*-actin signal. The data are expressed as the mean ± SEM. *n* = 6 for each group. A total of 6 samples were randomly selected from each group to perform western blotting. ^∗∗^*P* < 0.01 and ^∗∗∗^*P* < 0.001 vs. the Ad-GFP-sham group. CTRP9: C1q/TNF-related protein 9; PAH: pulmonary arterial hypertension; Ad-CTRP9: adenovirus-CTRP9; p38-MAPK: p38-mitogen-activated protein kinase; ERK: extracellular regulated protein kinase.

To determine whether CTRP9 affects the AKT pathway, the AKT, p38-MAPK, and ERK protein levels were assessed in the lung tissues of the rats. As expected, no significant difference was noted in the levels of phosphorylated AKT, p38-MAPK, and ERK between the Ad-GFP and Ad-CTRP9 groups (Figures 5(a) and 5(b)). However, following shunt placement, PAH rats treated with CTRP9 vector demonstrated significantly increased levels of phosphorylated AKT, p38-MAPK, and ERK, indicating that CTRP9 protected against PAH by activating the AKT pathway (Figures 5(a) and 5(b)).

## 4. Discussion

In the present study, CTRP9 reduced pulmonary artery remodeling by inhibiting SMC proliferation. RVSP and the inflammatory response of pulmonary and endothelial cells were also reduced, preventing and partially reversing arteriovenous shunt-induced PAH in rats. The molecular and cellular experiments further proved that CTRP9 downregulated the expression levels of TNF-*α* and MCP-1, while reducing the inflammatory infiltration of tissues and cells and mitigating the injury of pulmonary vascular endothelial cells via an increase in AKT phosphorylation levels *in vivo* and *in vitro*.

Arteriovenous malformations (AVMs) are composed of a complex vascular network, which is characterized by the direct connection of arteries that supply blood and veins providing drainage without a normal capillary network [18]. Following the formation of AVMs, the artery in the tissues is prone to hemorrhage and rupture, which may cause morbidity or death to the patients [19]. AMVs exist in major organs of the body but are mainly present in the brain, liver, and lung [20–22]. As previously reported, pulmonary artery malformation is a type of hereditary and hemorrhagic telangiectasia that directly communicates between the pulmonary artery and pulmonary vein without capillary intervention [21]. Hypoxemia and paradoxical emboli are complications of pulmonary artery malformation [23]. The arteriovenous shunt, monocrotaline injury, and chronic hypoxia exposure models are three classical animal models of PAH [16, 24–26]. The establishment and use of these animal models provide essential tools for the study of the large and complex processes of pulmonary artery malformation. In the present study, CTRP9 effectively alleviated PAH and other complications caused by arteriovenous shunts in rats. Moreover, the data demonstrated that in this model, arteriovenous shunt-induced PAH exhibited no effects on the body weight and the weights of the main organs, which was consistent with the models of chronic hypoxia exposure and monocrotaline injury in rats [27].

In order to assess the preventive and therapeutic effects of CTRP9 prior to modeling, CTRP9 was administered before establishment of the PAH model. If the animal model is established following CTRP9 overexpression, the therapeutic effect is not apparent and there may be no significant difference compared with the control group, which has suffered from irreversible damage to the lung [28]. In the present study, a PAH animal model was established to explore the beneficial effects of CTRP9 on the circulation system. The experiments were mainly focused on the pulmonary pathology caused by PAH. However, the cardiac failure caused by PAH was not the main focus of investigation in these experiments. Based on previous reports [29], heart damage will be determined and more attention should be paid on the development of PAH animal models in future studies.

Overexpression of CTRP9 inhibited the proliferation of SMC cells in the walls of the pulmonary vessels, while the intima and media were not readily distinguished in the shunt group following infection with the Ad-GFP. These findings are contradictory with the previous study performed by our research team [14]. In addition, the degree of stenosis of the vascular cavity in the vascular lumen was higher in the PAH models infected with Ad-CTRP9 compared with that noted in the Ad-GFP group. In addition, the damage of endothelial cells was reduced significantly, which was confirmed by a previous *in vitro* study [13]. In summary, the data indicated that CTRP9 alleviated intimal thickening of the pulmonary artery in the PAH model used.

Increased pulmonary arterial pressure and pulmonary vascular remodeling can lead to severe inflammatory response in the lung tissues [30]. The perivascular tissue of the pulmonary artery and the systemic inflammatory response lead to the occurrence and aggravation of vascular injury in patients with PAH [31]. In the current study, the data indicated that CTRP9 inhibited the expression of inflammatory factors in the lung tissues of PAH rats, which was consistent with previous studies conducted on several diseases, including myocardial infarction [10], retinopathy [32], asthma [33], type 2 diabetes mellitus [34], and atherosclerosis [11]. Notably, it has been proven by numerous studies that TNF-*α* is recognized as a proinflammatory cytokine that exhibits potent modulatory effects on pulmonary circulation [35–37]. Treatment with CTRP9 can significantly reduce the increased TNF-*α* expression (protein and mRNA) in PAH. In addition, macrophages, as resident cells of human tissues, have the ability to initiate and regulate the immune response during the periods of pathogen infection and tissue damage and develop specific phenotypes and functions in different tissues, which is helpful for maintaining tissue homeostasis [38]. In the current study, F4/80, which is a unique molecular marker of murine macrophages, was highly expressed in the lungs of rats with arteriovenous shunt-induced PAH. However, CTRP9 blocked the mRNA transcription of F4/80 and relieved the abnormal injury to the pulmonary endothelial cells in the shunt group, which was supported by the research team of Ghatnekar and his colleagues [39].

Under certain pathophysiological conditions, pulmonary injury in PAH is partially caused by apoptosis. It has been shown that miR-371b-5p inhibits endothelial cell apoptosis in a monocrotaline-induced PAH animal model [40]. Moreover, in a hypoxia-induced PAH model, silencing of the expression of the bone morphogenetic protein receptor type 2 impaired apoptosis by the selective transcriptional cleavage of the Bcl-x transcript in the lung and in smooth muscle endothelial cells [41]. Furthermore, fibroblast growth factor 21 significantly attenuated hypoxia-induced apoptosis and dysfunction of human endothelial cells by alleviating the aforementioned changes in the endoplasmic reticulum stress-dependent signaling pathways [42]. In the present study, overexpression of CTRP9 reduced caspase-8 protein expression and increased Bcl-2 protein expression in the shunt group, which was consistent with a previous study performed in PAH models [43].

Pulmonary hypertension is characterized by remodeling of the extracellular matrix, abnormal expression of proteolytic enzymes, increased deposition and cross-linking of collagen, and destruction of the elastic layer [44]. Extracellular matrix remodeling is caused by an imbalance in proteolytic enzyme expression and secretion during endothelial cell dysfunction and inflammatory responses [45]. MMPs comprise a specific type of proteolytic enzymes that play an important role in the regulation of extracellular matrix formation, cell migration, and cell growth. MMP-2 and MMP-9 enzymes, which are members of the matrix metalloproteinase family, participate in the transformation of type IV collagen and promote the migration and proliferation of SMCs [46]. The results of the present study indicated that overexpression of CTRP9 inhibited significantly MMP-2 and MMP-9 protein expression in the shunt group, suggesting that the protective effect of CTRP9 against PAH affected collagen deposition and delayed pulmonary endothelial cell remodeling. Interestingly, CTRP9 promoted the MMP2 protein expression, in contrast, decreased the MMP9 protein expression in the sham group compared to that in the control group. This new discovery will be explored in the future, which did not provide a profound discussion in the current experiment.

AKT signaling can regulate the balance between cell viability and apoptosis and maintain cell homeostasis by further controlling cell survival, apoptosis, growth, and glycogen metabolism [47]. Phosphorylated AKT promotes cell survival by antagonizing the apoptotic cascade signaling pathway following tissue injury. The activation of AKT has been shown to inhibit the transdifferentiation of pulmonary artery endothelial cells and the proliferation of pulmonary artery SMCs [48, 49]. Using animal models, the current study demonstrated that PAH caused the abnormal expression of extracellular matrix proteinases in rat lung tissues, indicating that vascular endothelial cell dysfunction led to an inflammatory response and induction of apoptosis. In Figure 5(b), overexpression of CTRP9-induced p-AKT protein expression increased significantly and the ratio of p-AKT/AKT was also elevated. According to your statement, we revised the sentence of discussion and prominent effect of activating the AKT signaling pathway. *In vitro*, cytobiology experiments indicated that CTRP9 alleviated the endothelial cell injury induced by TNF-*α* by promoting the phosphorylation of AKT [13]. Moreover, it was further shown that CTRP9 reduced vascular endothelial injury and inhibited pulmonary arterial pressure vascular remodeling following arteriovenous shunting.

In conclusion, the results of the present study demonstrated that CTRP9 improved vascular remodeling in an animal model of PAH induced by arteriovenous shunts, notably by alleviating inflammation, apoptosis, and extracellular matrix damage in the perivascular tissue of the pulmonary artery. In addition, the establishment of PAH was explored by the arteriovenous shunt, which simulated the disease mechanisms of congenital vascular malformation and provided a theoretical and experimental basis for clinical treatment. Furthermore, CTRP9 regulated the inflammatory response and dysfunction of vascular endothelial cells by increasing the phosphorylation of AKT and significantly improved pulmonary arterial remodeling and vascular stenosis in rats. These findings provide strong experimental evidence for the molecular mechanism by which CTRP9 protects against PAH *in vivo*. Therefore, the results of the present study provided novel insight into the mechanisms underlying arteriovenous shunt-induced PAH.

## 5. Study Limitation

In the present study, we established a PAH animal model to explore the beneficial effects of CTRP9. We mainly focused on pulmonary pathology caused by PAH. The cardiac failure caused by PAH is not the key point of our investigation in the present study. We mainly focus on pulmonary pathology caused by PAH and the therapeutic effects after being administrated by CTRP9 by the tail vein. In Figure 2(e), we observed the thickness of pulmonary artery smooth muscle decreased significantly by treatment of CTRP9 compared to the GFP group in shunt operation. Hence, overexpression of CTRP9 effectively alleviated the pathological symptoms of PAH caused by arteriovenous shunt. In order to clarify the molecular mechanism of CTRP9, we analyzed MAPK signaling pathway molecules and found that the level of phosphorylation of AKT and p38 MAPK increased significantly after being administrated by CTRP9 in the shunt group. Therefore, CTRP9 relieves the PAH by activating the MAPK/Akt signaling pathway. Additionally, based on the reviewers' suggestion, we will determine and focus on the heart tissue damage caused by PAH in animal models in future studies. Furthermore, 10 rats were used from each group and their body weight and arterial pressures were measured. Considering the number of animals and multiple groups, we randomly selected 6 samples from each group to perform the western blotting and RT-qPCR. Therefore, the number of animals was not consistent in the different test items. Thirdly, specific experiments were performed to explore the molecular mechanism of CTRP9 on the PAH animal model in vivo. The effects noted when inhibitors of key signaling molecules were used, such as AKT or MAPK, are aimed at identifying the interaction of these signaling pathways with PAH and CTRP9.

## Data Availability

The data used to support the findings of this study are available from the corresponding author upon request.

## Abbreviations

Ad: Adenovirus
AKT: AKT serine/threonine kinase 1
AVMs: Arteriovenous malformations
Bcl-2: b-cell lymphoma 2
CTRPs: C1q/TNF-related protein families
DMEM: Dulbecco's modified eagle medium
ED: Vascular external diameter
ERK: Extracellular regulated protein kinases
FBS: Fetal bovine serum
GFP: Green fluorescent protein
H&E: Hematoxylin and eosin
ID: Internal diameter
IL-6: Interleukin 6
LA: Lumen area
MT: Medial thickness
MAPK: Mitogen-activated protein kinase
MCP-1: Monocyte chemotactic protein 1
mPAP: Mean pulmonary arterial pressure
MMP: Matrix metallopeptidase
PVDF: Polyvinylidene fluoride
PAH: Pulmonary artery hypertension
RVSP: Right ventricular systolic pressure
SMCs: Smooth muscle cells
SD: Sprague-Dawley
TNF-*α*: Tumor necrosis factor alpha
WT: Wall thickness.

## Supplementary Materials

Supplementary Materials Supplementary Table 1: sequences of primers used for qRT-PCR analysis in rat lung tissue.
