# Supplementary material for: CTRP9 Mitigates the Progression of Arteriovenous Shunt-Induced Pulmonary Artery Hypertension in Rats
Source: Cardiovasc Ther. 2021 Nov 10;2021:4971300. doi: 10.1155/2021/4971300 (PMC8598355; doi:10.1155/2021/4971300)
Supplement: Supplementary Materials — Supplementary Table 1: sequences of primers used for qRT-PCR analysis in rat lung tissue. [file 4971300.f1.docx]

Supplementary Table 1: Sequences of primers used for qRT-PCR analysis in rat lung tissue.

| Gene | Forward (5’-3’) | Reverse (5’-3’) |
| --- | --- | --- |
| CTRP9 | ACTCCAGGTCACAATGGCCTAC | GAGCCTGGATCACCTTTGATG |
| MCP-1 | AGCAGCAGGTGTCCCAAAGA | GTGCTGAAGACCTTAGGGCAGA |
| F4/80 | CTTTGGCTATGGGCTTCCAGTC | GCAAGGAGGACAGAGTTTATCGTG |
| IL-6 | CCACTTCACAAGTCGGAGGCTTA | CCAGTTTGGTAGCATCCATCATTTC |
| IL-10 | TGAATTCCCTGGGTGAGAAG | TCACTCTTCACCTGCTCCACT |
| IL-18 | GACTCTTGCGTCAACTTCAAGG | CAGGCTGTCTTTTGTCAACGA |
| TNF-α | TATGGCCCAGACCCTCACA | GGAGTAGACAAGGTACAACCCATC |
| β-Actin | GCGGCATCCACGAAACTAC | TGATCTCCTTCTGCATCCTGTC |
